# Sleep problems among children with Fetal Alcohol Spectrum Disorders (FASD)- an explorative study

**DOI:** 10.1186/s13052-021-01056-x

**Published:** 2021-05-17

**Authors:** Katarzyna Anna Dylag, Bożena Bando, Zbigniew Baran, Paulina Dumnicka, Katarzyna Kowalska, Paulina Kulaga, Katarzyna Przybyszewska, Jakub Radlinski, Sylvia Roozen, Leopold Curfs

**Affiliations:** 1 St. Louis Children Hospital, Strzelecka 2, 31-503, Kraków, Poland; 2 National Research Institute for Tuberculosis and Lung Diseases, Rabka Branch, Prof. Jana Rudnika 3B, 34-700 Rabka-Zdrój, Poland; 3 Jagiellonian University Medical College, Department of Medical Diagnostics, Medyczna 9, 30-688, Kraków, Poland; 4 Governor Kremers Centre, Maastricht University Medical Centre, PO Box 616 6200, MD Maastricht, The Netherlands

**Keywords:** Fetal alcohol spectrum disorders, Prenatal alcohol exposure, fetal alcohol syndrome, Partial fetal alcohol syndrome, alcohol related neurodevelopmental disorders, Sleep disorders

## Abstract

**Background:**

Fetal alcohol spectrum disorders (FASD) is a group of conditions resulting from prenatal alcohol exposure (PAE). Patients with FASD experience a variety of neuropsychological symptoms resulting from central nervous system impairment. Little is known about sleep disorders associated with PAE. The objective of this study was to investigate sleep problems related to FASD.

**Methods:**

Forty patients (median age 8 years (6; 11)) diagnosed with FASD and forty typically developing children (median age 10 years (8; 13)) were recruited for the 1st phase of the study. In the 1st phase, the screening of sleep problems was performed with Child Sleep Habit Questionnaire (CSHQ) filled in by a caregiver. Those of the FASD group who scored above 41 points were qualified to the 2nd phase of the study and had an in-lab attended polysomnography (PSG) performed. The measurements consisted of electroencephalogram, electrooculograms, chin and tibial electromyogram, electrocardiogram, ventilatory monitoring, breathing effort, pulse oximetry, snoring and body position. Their results were compared to PSG laboratory reference data.

**Results:**

The number of participants with sleep disturbances was markedly higher in the FASD group as compared to typically developing children (55% vs. 20%). The age-adjusted odds ratio for a positive result in CSHQ was 4.31 (95% CI: 1.54–12.11; *p* = 0.005) for FASD patients as compared to the control group. Significant differences between the FASD as compared to the typically developing children were observed in the following subscales: sleep onset delay, night wakings, parasomnias, sleep disordered breathing, and daytime sleepiness. Children from the FASD group who underwent PSG experienced more arousals during the sleep as compared with the PSG laboratory reference data. The respiratory indices in FASD group appear higher than previously published data from typically developing children.

**Conclusion:**

The results support the clinical observation that sleep disorders appear to be an important health problem in individuals with FASD. In particular distorted sleep architecture and apneic/hypopneic events need further attention.

## Background

Fetal alcohol spectrum disorders (FASD) is an umbrella term used to describe a spectrum of conditions resulting from prenatal alcohol exposure (PAE) including: fetal alcohol syndrome (FAS), partial fetal alcohol syndrome (pFAS), alcohol related neurodevelopmental disorder (ARND) and alcohol related birth defect (ARBD) [1]. Characterized by high global prevalence [2, 3], FASD is one of the most common neurodevelopmental conditions.

An impaired brain function associated with PAE is the main clinical concern among patients with FASD [4]. The variety of brain functions or domains can be affected including domains such as: cognition [5, 6]- especially executive functions [7] and social skills [8]. However, each individual with FASD can present with a different clinical picture. Many patients experience not only the neurobehavioral issues but also disorders of urination [9, 10], defecation [9, 10], nutrition [11, 12] and sleep, also known as “neglected problems”. It is now well established that sleep disorders are an important concern among children with neurodevelopmental conditions [13–15]. Sleep disorders affect daytime performance of children and can influence the diagnostic process creating a complex clinical picture [16–19]. Clinical experience, case reports and qualitative studies [6, 20, 21] suggest that sleep disorders are a major complaint among individuals with FASD.

Several studies used quantitative, questionnaire methods to assess the sleep problems among individuals with FASD. Results of this studies illustrate that the sleep disorders are more prevalent in patients with FASD as compared to typically developing children [13, 22–25].

The reports including objective sleep assessment in patients with FASD are scarce [13, 22, 23]. Pesonen at al [26], Wengel at al [27], and Mughal et al. [18] demonstrated sleep disturbances among individuals with FASD using actigraphy. Goril at al [28] and Chen et al. [29] examined children with FASD with polysomnography and pointed at some specific problems such as increased sleep fragmentation. However, due to the lack of a control group in the first study and small sample size (5 patients) of the latter, their data can be considered exploratory.

Given the little attention paid so far to sleep, the aim of this study was to characterize sleep in children with FASD in depth.

## Material and methods

**Fig. 1:**
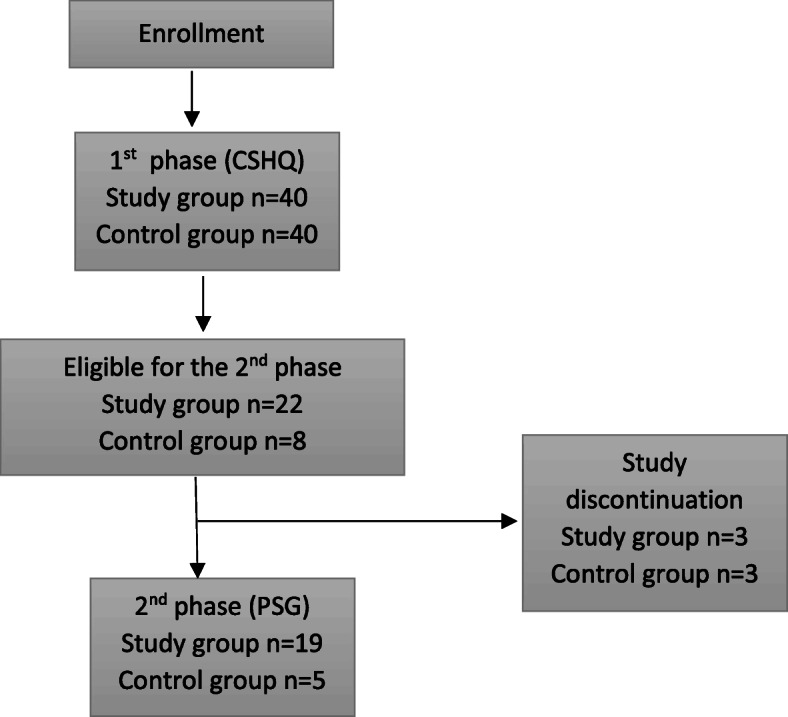
Study diagram

The study was conducted as a prospective study in the years 2018–2020. The study included children diagnosed with FASD and a control group and consisted of two phases. In the 1st phase, the screening of sleep problems was performed with Child Sleep Habit Questionnaire (CSHQ) filled in by a caregiver. The subjects with positive CSHQ results were qualified to the 2nd phase of the study, an in-lab attended polysomnography (PSG) (Fig. 1).

A written consent was obtained by the researchers from the parent/caregiver. A separate consent was obtained from the children above 12 years of age. Children younger than 12 were informed about the study and all their questions about the study were answered. The study protocol was approved by the regional ethical committee of the Regional Board of Physicians (approval number: 123/kbl/oil/2018).

### Subjects

The study group consisted of 40 patients of a FASD Diagnostic Center functioning in a regional hospital. During the final diagnostic visit in the clinic, when the diagnostic process is summarized, the patients were offered the participation in the study by an independent researcher. The inclusion criteria were: the FASD diagnosis established by a multidisciplinary team according to Hoyme 2016 criteria [3], and age 3–17 years at the moment of the enrollment. The exclusion criteria were: a comorbid pulmonary disease causing a distorted sleep (severe asthma, cystic fibrosis), another birth defect, or a known seizure disorder. 40 consecutive patients eligible for the study during the study period were offered the participation and all of them agreed to participate in the study. No patient from the FASD met the exclusion criteria.

The control group was formed from patients of the Gastroenterology Department of the same hospital diagnosed with a small intestine bacterial overgrowth (SIBO) and successfully treated. The participation was offered during a follow-up visit, after the patients were asymptomatic regarding abdominal pain for at least 3 months. Patients in age 3–17 years were included. The exclusion criteria for the control group were: confirmed or unknown PAE, microcephaly, height/weight below 10th percentile, presence of facial dysmorphic features characteristic for FASD (smooth philtrum, narrow upper lip, short palpebral fissure), comorbid pulmonary disease causing distorted sleep (severe asthma, cystic fibrosis), comorbid neurologic or neurodevelopmental condition causing a distorted sleep (e.g. attention deficit hyperactivity disorder, spinal muscular atrophy). 40 consecutive patients eligible for the study during the study period were offered the participation and all of them agreed to participate in the study. No control subject met the exclusion criteria.

Twenty-two patients from the FASD group and 8 from the control group (everybody with TSC > 41) were eligible for the second phase of the study, however, 3 patients both from the FASD group and from the control group discontinued the participation due to a caregiver’s refusal. (Fig. 1) Retrospective data obtained at the polysomnography laboratory were used to form a PSG laboratory control group (*n* = 105) in order to enable the comparison of the sleep architecture indices of the which values may be laboratory specific. The PSG laboratory control group was composed of the typically developing children who had the PSG performed mainly due to the request of their caregivers or primary care physicians. The inclusion criteria consisted of: the lack of comorbidities or genetic syndromes and the negative result of PSG in term of sleep disordered breathing (a number of breathing events per hour of sleep less than one). The group was treated as a population sample representative for the PSG laboratory and reference intervals for sleep related parameters were calculated after removal of 10% extreme values.

## Methods

### CSHQ

Parents/caregivers of the patients eligible for participation in the study were asked by an independent researcher to fill in the Child’s Sleep Habits Questionnaire (CSHQ) – an abbreviated form [30]. This questionnaire is a well-established tool for screening pediatric sleep problems [30–33]. It consists of 33 questions covering the following aspects of sleep: bedtime resistance, sleep onset delay, sleep duration, sleep anxiety, night wakings, parasomnias, sleep distorted breathing and daytime sleepiness. According to the questionnaire instruction, the questions about child’s habits were answered by the caregiver based on their observation during the last typical week (if the last week was somehow unusual, the last usual week is used). The caregiver was asked to answer usually (if something is occurring 5 or more times in a week), sometimes (2–4 times in a week) or rarely (never or 1 time during a week). Based on the answers of the caregivers, a total sleep disturbance score was calculated according to the questionnaire instruction [30]. The cutoff was established at 41 points [30]. Patients whose score was above the cutoff were contacted by phone and asked to participate in the second phase of the study.

### Polysomnography

In the second phase of the study, the in-lab attended polysomnography (PSG) was performed (Alice 6 Philips Respironics) according to American Academy of Sleep Medicine (AASM) recommendations [34].

The measurements consisted of electroencephalographic channels (O1/A2, O2/A1, C3/A2, C4/A1, F3/A2, F4/A1), left and right electrooculograms, chin electromyogram, left and right tibialis electromyogram, electrocardiogram, ventilatory monitoring using thermal and pressure sensors, breathing effort monitoring using thoracic and abdominal belts, pulse oximetry, snoring and body position monitoring. Hooking-up of the subjects was performed directly before the start of the measurement, usually between 7 and 9 p.m. The subjects were woken up around 6 a.m. All recordings were manually scored, sleep staging and scoring breathing events were performed using pediatric criteria [34]. Sleep-stage percentages were calculated on the basis of the total sleep time (TST) and for wake periods as percentage of time in bed (TIB), i.e. the time measured from lights out to lights on. Sleep efficiency was calculated as the sleep stages percentage of TIB. Sleep latency was defined as a time from lights-off to the beginning of the first epoch considered as sleep (N1, N2, N3, REM). Stage shift means a change from one sleep stage to another. Arousals and breathing events were presented as common indices – the number of events per hour of sleep. Respiratory disturbance index (RDI) were calculated as the number of respiratory events (apneas all types, hypopneas, respiratory effort related arousals – RERA’s) per hour of sleep. In the case of our study RDI effectively equals to apnea hypopnea index due to the lack of RERA’s observations in examined groups.

### Statistical analysis

The number of patients and percentage of the respective group (as specified in the Results) were reported for the categories. Contingency tables were analyzed with Pearson chi-squared test. Age-adjusted logistic regression was used to confirm the difference in CSHQ results between FASD patients and controls. Continuous variables were exposed as median and lower and upper quartiles because not all the data were normally distributed. For comparison with previous sleep studies, selected parameters were exposed as a mean and standard deviation. Wilcoxon (Mann-Whitney) rank sum test was used to examine differences between two groups, for a greater number of groups, Kruskal-Wallis test was applied. The association between continuous variables were examined using a linear regression model. Calculations were performed using R environment for statistical computing [35] (Microsoft R Open 3.5.3) and Statistica 12.0 (StatSoft, Tulsa, OK, USA).

## Results

### Sample characteristics

The median age of the FASD group was 8 years (6; 11), the group consisted of 19 males and 21 females (Table 1). Twelve percent of the patients were with at least one of the biological parents, 35% were in a foster care, whilst 52% were in adoptive families. Within FASD, the most common diagnosis was ARND (42%), followed by pFAS (32%) and FAS (25%). When compared with the control group, patients from the FASD group had an equal sex distribution and body mass index (BMI) but were slightly younger (Table 1).

**Table 1:** Characteristics of studied patients with FASD and control subjects

	FASD patients (*n* = 40)	Control group (n = 40)	p
Diagnosis:			
FAS, n (%)	10 (25)	NA	NA
pFAS, n (%)	13 (32)		
ARND, n (%)	17 (42)		
Age, years	8 (6; 11)	10 (8; 13)	0.02
Male sex, n (%)	19 (47)	18 (45)	0.8
Family:			
Biological, n (%)	5 (12)	40 (100)	< 0.0001
Foster, n (%)	14 (35)	0	
Adoptive, n (%)	21 (52)	0	
Weight, kg	24 (17; 33)	30 (21; 42)	0.04
Height, m	129 (112; 139)	138 (123; 153)	0.02
BMI, kg/m^2^	15.2 (13.9; 17.6)	15.9 (14.8; 18.0)	0.2

### CSHQ

**Fig. 2:**
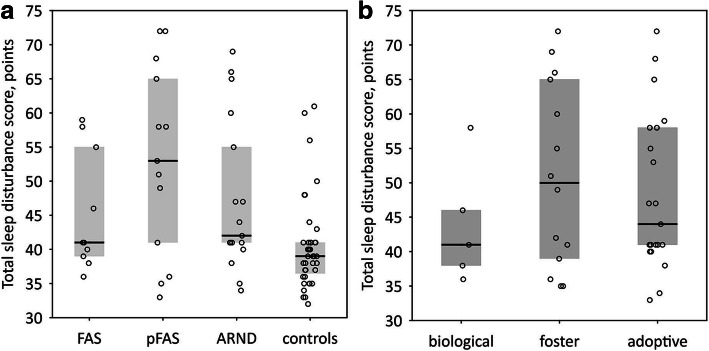
Total sleep disturbance score in the studied children with FASD according to diagnosis in comparison with neurotypical controls (**a**) and according to the type of a family (**b**). There were no significant differences between FAS, pFAS and ARND patients (*p* = 0.4) and between FASD patients in biological, foster or adoptive families (*p* = 0.6). The data are shown as median (central line), interquartile range (box) and raw data (points)

The number of participants with sleep disturbances, i.e. total sleep disturbance score above the cutoff, was markedly higher in the study group as compared to controls (55% vs. 20%, Table 2). The age-adjusted odds ratio for a positive result in CSHQ (i.e. total sleep disturbance score > 41) was 4.31 (95% confidence interval: 1.54–12.11; *p* = 0.005) for FASD patients as compared to the control group. Among children with FASD, BMI was not associated with the total sleep disturbance score > 41 (*p* = 0.09 after adjustment for age). There were no significant differences in CSHQ results (subscales and total sleep disorder score) between children diagnosed with FAS, pFAS and ARND (Fig. 2a). Moreover, the diagnosis was not significantly associated with the percentage of positive CSHQ results [4 (40%) of patients with FAS, 9 (69%) of patients with pFAS, and 9 (53%) of patients with ARND; *p* = 0.4]. There were no significant differences in CSHQ results between FASD children depending on the type of a family (Fig. 2b). The type of a family was not significantly associated with the positive result in CSHQ among patients with FASD [2 (40%) of children in biological families, 9 (64%) in foster families, and 11 (52%) in adoptive families; *p* = 0.6]. Significant differences between the study and control groups were observed in the following subscales: sleep onset delay, night wakings, parasomnias, sleep disordered breathing, and daytime sleepiness (Table 2).

**Table 2:** The results of CSHQ in FASD patients and controls

Children’s Sleep Habits Questionnaire:	FASD patients (n = 40)	Control group (n = 40)	p
Bedtime resistance, points	8 (6; 10)	7 (7; 8.5)	0.2
Sleep onset delay, points	1 (1; 2.5)	1 (1; 1)	0.02
Sleep duration, points	3 (3; 4)	3 (3; 4)	0.3
Sleep anxiety, points	5 (4; 6.5)	4 (4; 5.5)	0.3
Night wakings, points	4 (3; 5)	3 (3; 4)	0.01
Parasomnias, points	9 (8; 11.5)	7 (7; 8)	0.0003
Sleep disordered breathing, points	3 (3; 5)	3 (3; 3)	0.02
Daytime sleepiness, points	11 (7.5; 13.5)	8 (7; 9)	0.0009
Total sleep disturbance score, points	45 (40; 58)	39 (36.5; 41)	0.0007
Total sleep disturbance score > 41 points, n (%)	22 (55)	8 (20)	0.001
Polysomnography, n (%)	19 (47)	5 (12)	0.0006

### Polysomnography

Children from the FASD group had a distorted sleep architecture in comparison with the patients from the PSG laboratory control group (Tables 3 and 4). The number of stage shifts was greater, the proportion of time spent in the N1 stage of sleep was higher and the proportion of the summary time of N3 and REM was significantly lower among the patients with FASD than among the patients from the PSG laboratory control group (Table 3). Both children from the FASD group and children from the control group experienced more arousals during the sleep than the children from the PSG laboratory control group (Tables 3 and 4). Both in the FASD group and in the 5 children from the control group, the respiratory indices were higher as compared to the previously published data for a normal population (Table 5). There was no association between the arousals index and RDI in the FASD group (R^2^ < 0.01, *p* = 0.73). The similar lack of relationship was observed between the obstructive apnea index and central apnea index (R^2^ < 0.18, *p* = 0.07) and between the hypopnea index and central apnea index (R^2^ = 0.14, *p* = 0.62) in the FASD group.

**Table 3:** Polysomnography results

	FASD patients (*n* = 19)	Control group (*n* = 5)	PSG laboratory control group (n = 105)	p
Age, years	7.1 (5.5; 9.3)	7.5 (6.7; 8.7)	6.5 (4.9; 8.9)	0.7
Male sex, n (%)	8 (42)	3 (60)	66 (63)	0.2
Aquisition time, min	594 (578; 607)	582 (570; 584)	591 (572; 603)	0.4
TIB, min	548 (530; 569)	538 (529; 544)	543 (524; 563)	0.8
TST, min	464 (434; 507)	449 (446; 480)	466 (433; 494)	0.9
SPT, min	526 (502; 543)	512 (509; 534)	517 (494; 537)	0.8
Sleep efficiency, %TIB	86.4 (78.4; 91.1)	82.6 (81.4; 82.9)	86.1 (79.7; 92.8)	0.8
Sleep latency, min	22.5 (7.4; 28,6)	18.9 (11.0; 30.2)	16.0 (5.5; 26.1)	0.5
Stage shifts, n	86 (64; 97)	75 (57; 98)	67 (54; 83)	0.03 ^a^
W, %SPT	9.7 (3.4; 14.7)	12.3 (11.9; 16.5)	9.8 (4.2; 15.2)	0.6
N1, %TST	4.3 (2.6; 5.7)	3.2 (3.0; 3.7)	2.4 (1.8; 3.6)	0.005 ^a^
N2, %TST	53.7 (47.6; 58.0)	54.4 (47.4; 57.3)	50.4 (46.2; 53.5)	0.1
N3, %TST	27.5 (22.1; 31.5)	26.8 (24.6; 29.8)	29.0 (25.0; 32.5)	0.2
REM, %TST	15.1 (14.5; 19.4)	17.8 (17.4; 19.9)	17.6 (14.9; 20.6)	0.4
N3 + REM, %TST	42.5 (39.5; 46.5)	42.5 (37.0; 50.5)	46.7 (42.1; 50.5)	0.03 ^a^
Arousal index, events/h	6.9 (5.9; 8.5)	8.5 (7.7; 9.6)	5.1 (4.1; 6.2)	< 0.0001 ^a,b^
Total apnea index, events/h	1.0 (0.7; 1.8)	2.1 (0.8; 5.4)		0.3
Central apnea index, events/h	1.0 (0.5; 1.8)	2.0 (0.8; 4.3)		0.3
Obstructive apnea index, events/h	0 (0; 0.2)	0 (0; 0.6)		0.6
Mixed apnea index, events/h	0 (0; 0.1)	0.1 (0; 0.1)		0.6
Hypopnea index, events/h	0.5 (0; 1.2)	0.8 (0.2; 1.6)		0.5
Obstructive apnea hipopnea Index, events/h	0.7 (0.2; 1.3)	0.98 (0.25; 2.75)		0.6
Respiratory disturbance index, events/h	1.7 (1.0; 3.2)	2.9 (0.8; 7.0)		0.6
Minimal SaO_2_, %	91 (90; 92)	92 (86; 92)		0.5
Average SaO_2_, %	97 (96; 97)	97 (96; 97)		1
Oxygen desaturation index, events/h	1.8 (0.9; 2.9)	2.2 (0.8; 8.1)		0.5

^a^ significant difference between FASD patients and the PSG control group

^b^ significant difference between the control group and the PSG control group

**Table 4:** Polysomnography results in FASD patients and the control group exceeding the reference intervals based on the PSG laboratory control group

	Reference interval	Number (%) of patients with result above or below reference interval			
		FASD patients (n = 19)		Control group (n = 5)	
		Below	Above	Below	Above
Sleep efficiency, %	≥63.38	0	NA	0	NA
Sleep latency, min	0.6–85.2	0	1 (5)	0	0
Stage shifts, n	45.2–108.4	0	1 (5)	0	0
W, %SPT	1.42–28.34	1 (5)	0	0	0
N1, %TST	0.5–7.9	0	2 (11)	0	1 (20)
N2, %TST	39–62.4	1 (5)	0	0	0
N3, %TST	19.8–43.7	3 (16)	0	1 (20)	0
REM, %TST	6.1–27.4	0	0	0	0
Arousal index, events/h	≤7.7	NA	7 (37)	NA	3 (60)

**Table 5:** Comparison selected PSG indices with previous sleep studies in general children population (mean ± standard deviation)

	Current study	Montgomery- Downs [36]	Montgomery-Downs [36]	Goodwin [37]
Age, range, years	2.9–12.6	3.2–5.9	6.0–8.6	6–11
N	19	153	388	480
TST, min	464 ± 50.4	475.2 ± 42.0	471.6 ± 43.7	487 ± 79.7
Sleep efficiency, %TIB	84.8 ± 7.9	90.0 ± 7.0	89.3 ± 7.5	89.8 ± 5.8
Sleep latency, min	24.7 ± 25.7	24.1 ± 25.6	23.0 ± 25.3	18.5 ± 21.0
W, %TIB	15.2 ± 7.9	9.4 ± 7.3	8.1 ± 7.1	
N1, %TST	4.5 ± 2.5	6.6 ± 4.8	7.1 ± 5.5	4.6 ± 3.3
N2, %TST	52.5 ± 6.3	41.6 ± 7.1	46.1 ± 8.5	52.0 ± 6.1
N3, %TST	26.8 ± 6.2	S3: 6.6 ± 2.6 S4: 21.6 ± 6.2	S3: 5.5 ± 2.9 S4: 18.5 ± 6.6	21.9 ± 6.9
REM, %TST	16.2 ± 4.0	23.6 ± 4.8	22.2 ± 5.2	21.5 ± 5.0
Arousal index, events/h	7.4 ± 2.2	9.0 ± 3.4	9.5 ± 5.3	
Total apnea index, events/h	1.8 ± 1.8	0.86 ± 0.75	0.50 ± 0.52	
Central apnea index, events/h	1.6 ± 1.5	0.82 ± 0.73	0.45 ± 0.49	0.28 ± 0.84
Obstructive apnea index, events/h	0.2 ± 0.3	0.03 ± 0.1	0.05 ± 0.11	0.02 ± 0.27
Mixed apnea index, events/h	0.1 ± 0.2	0.01 ± 0.05	0.01 ± 0.06	
Hypopnea index, events/h	1.3 ± 3.0	0.03 ± 0.07	0.10 ± 0.18	
Apnea hypopnea index, events/h	3.2 ± 4.4	0.90 ± 0.78	0.68 ± 0.75	0.90 ± 1.91
Minimal SaO_2_, %	90.1 ± 3.1	92.7 ± 4.5	92.6 ± 3.6	
Average SaO_2_, %	96.4 ± 1.2			

## Discussion

According to our results, every second child with FASD experiences sleep disorders. This finding is consistent with the result of the research published by Goril et al. [28] who estimated the prevalence of sleep disorders among individuals with FASD to 58%. On the other hand, Chen et al. [29] established a prevalence rate at 85%. Although similarly to our study, Chen et al. used the CSHQ, they classified children with the borderline Total Score of 41 as having sleep disturbances whilst we, following the CSHQ instruction [30, 38], qualified only the results above 41 as positive. However, 7 children from the FASD group (17.5%) and 7 children from the control group (17.5%) had a Total Score equal with 41. Our subscale analysis revealed that sleep onset delay, night wakings, parasomnias, sleep disordered breathing and daytime sleepiness occur more frequently among individuals with FASD as compared to controls. These results are in general agreement with the data of Chen et al. [29] who reported an increased frequency of sleep problems in all 8 subdomains of CSHQ. Our observations are also in line with the findings of Wengel et al. [27] who indicated that patients with FASD complained about shortened sleep duration, night awakenings and parasomnias. Hayes et al. using a simple 3-question online questionnaire (asking about difficulty falling to sleep; difficulty staying asleep and/or frequent waking during the night; and waking early in the morning) established a frequency of sleep problems among individuals with FASD to 65,6% finding difficulty falling asleep being the most common complaint [24]. The reported frequency is similar to our findings, moreover, in our study the sleep onset delay was also more common among FASD patients than in the control group. Increased odds of sleep problems reported by us corroborates with the findings presented by Chandler-Mather at al. [25] who assessed sleep problems with a yes-no, 4-question questionnaire (“getting off to sleep at night”, “not happy to sleep alone”, “waking during the night”, and “restless sleep”).

Polysomnography data on FASD patients are scarce. Our findings match those published by Chen et al. [29], although we observed that not only obstructive but also central apneic events were more frequent among individuals with FASD. Also, our findings are in line with the report of Goril et al. [28] who indicated an increased sleep fragmentation in children with FASD and an increased predisposition to apneic/hypopneic events. Interestingly, the increased AHI was only observed in the young children in their sample. Moreover, Alvik et al., Troese et al. and Scher et al. [39–41] demonstrated that infants with PAE tend to present fragmented sleep and experience more arousals. This is in concordance with our findings, although these studies did not use PSG and the results cannot be directly compared to ours. A similar observation was documented by Volgin et al. [42] on an animal model of PAE.

In our study, sleep in FASD subjects was less stable than in the PSG laboratory control group which is mainly expressed in a greater number of stage shifts, an increased amount of N1 stage and a higher number of arousals. The lack of association between arousals and breathing events suggests that the subset of arousals has other than a respiratory cause. The comparison of our findings regarding sleep architecture to the general population samples (Table 5) is difficult due to inconsistencies in published pediatric population data [36, 37]. Montgomery-Downs et al. [36] reported the amount of N1 sleep higher than in our subjects but Goodwin et al. [37] reported it approximately at the same level. The amount of N3 in our patients with FASD is comparable to the Montgomery-Downs sample [36] but much higher than in the Goodwin sample [37]. Of note, the percentage of REM sleep is lower in our FASD group in comparison to both studies [36, 37]. Moreover, these results seem to be consistent with experimental data [43] of a decreased quantity of REM sleep in rats with PAE, however, this observation was only significant for female rats.

In our study, both FASD and control children examined by polysomnography had much more breathing events detected than reported in general population samples [36, 37]. It is clearly visible in all compared indices (Table 5). It must be acknowledged that the changes in the hypopnea definition which were introduced in version 2 of AASM manual published in 2018 [34] led to an increased number of detected hypopnea events. According to the authors’ knowledge, there are no published reports about how those recent changes affect hypopnea index results in children population. Still, the hypopnea index in our study is larger by an order of magnitude as compared to previously published population data [36, 37] which is more than a potential impact of the definition changes. Moreover, the central apnea index in our FASD group is greater by a factor of two, even though the current pediatric central apnea definition is more restrictive.

The cause of sleep problems among individuals with FASD remains uncertain. Prenatal alcohol exposure may cause structural defects of central nervous system [4, 44–46], corpus callosum anomalies being the most common finding [47–50]. It has been demonstrated that corpus callosum anomalies may result in sleep alteration [23], especially decreased REM sleep. This finding of decreased REM sleep is in accordance with what we observed in our FASD group. The potential mechanisms lying behind sleep problems in FASD include not only structural defects of some parts of the brain but also important dysfunction of neurotransmission [16]. Olateju et al. [51] established a relationship between PAE and the alterations of orexinergic and cholinergic neurons in brain structures responsible for cicardian rhythms. Moreover, the destructive influence of PAE, direct or secondary do the epigenetic mechanisms, on the neurons of the suprachiasmatic nuclei (SCN) responsible for the natural rhythms, was also postulated [52]. Chen et al. [53] suggested, using the animal model, that PAE affects expression of the genes responsible for the circadian function of β-endorphin neurons in the hypothalamus. On the other hand, Goril et al. [28] determined that children with FASD have an altered melatonin profile, which can affect the sleep cycle. The central apneic/hypopneic events likely result from the central nervous damage secondary to PAE, as it has been established that alcohol affects the neurons in the variety of experimental modes [4, 44–46, 54]. It is worth mentioning that obstructive apneic/hypopneic events were frequent among children with FASD, although none of the patients were obese. Nevertheless, the anatomical features connected to FASD – micrognathia or high-arched palate may result in the airway obstruction [55, 56]. It is also possible that sleep disturbances, especially parasomnias and difficulties falling asleep are secondary to the behavioral problems that children with FASD are facing [18, 57].

This is one of the first studies that comprehensively assess the sleep problems among children with FASD. The second phase of our study was the first attempt to objectively characterize sleep alterations present in children with FASD using polysomnography in a significant group of prospectively recruited patients. Nevertheless, several limitations of our study should be acknowledged. Due to organizational reasons, the enrollment to the both FASD and control groups was performed simultaneously which resulted in the negligible age difference between the groups. However, patients from both groups were within the age frame for CSHQ (3–17 years) and all differences observed between the groups were independent of age. CSHQ is a subjective sleep problems assessment. Only the patients screened by CSHQ were offered the objective sleep evaluation. The majority of parents in the study group were either foster or adoptive parents yet the patients from the control group were in biological families. It is well established that foster and adoptive parents of FASD children experience a lot of distress and they present a tendency to an overprotective attitude [24, 58] which can be a potential source of bias in the self-report.

Notwithstanding the limitations, the study offers the overview of the frequency and nature of sleep problems among individuals with FASD. As the results point to an increased frequency of sleep issues in FASD, further research is required to confirm and expand this finding. Moreover, further experimental investigations are needed to determine the exact mechanisms that contribute to this phenomenon as well as potential treatment options. From the clinical perspective it is important for the physicians and psychologists taking care for patients with FASD to include the question about the sleep quality in the history taking and try to address this issue.

## Conclusion

FASD is one of the leading preventable forms of neurodevelopmental disorders. The pathophysiological understanding of FASD is insufficient. The sleep problems are until now not well understood in relation to FASD. Our study supports previous observations that sleep problems can be a part of the clinical picture in FASD and that a distorted sleep architecture and apneic/hypopneic events can be present, what needs further studies.

## Data Availability

The datasets used and/or analyzed during the current study are available from the corresponding author on reasonable request.

## Abbreviations

AASM: American Academy of Sleep Medicine
ADHD: Attention Deficit Hyperactivity Disorder
AHI: Apnea–Hypopnea Index
ARND: Alcohol- Related Neurodevelopmental Disorder
CSHQ: Child’s Sleep Habits Questionnaire
FASD: Fetal Alcohol Spectrum Disorders
FAS: Fetal Alcohol Syndrome
PAE: Prenatal Alcohol Exposure
PFAS: Partial Fetal Alcohol Syndrome
PSG: Polysomngraphy
RDI: Respiratory Disturbance Index
REM: Rapid Eye Movement
RERA: Respiratory Effort Related Arousal
TIB: Time In ed.
TST: Total Sleep Time
